# Supplementary material for: Comparative Genomic Analysis of a Novel Vibrio sp. Isolated from an Ulcer Disease Event in Atlantic Salmon (Salmo salar)
Source: Microorganisms. 2023 Jul 2;11(7):1736. doi: 10.3390/microorganisms11071736 (PMC10385127; doi:10.3390/microorganisms11071736)
Supplement: Supplementary file 1 [file microorganisms-11-01736-s001.zip › Article 1- Supplementary Figures.pdf]

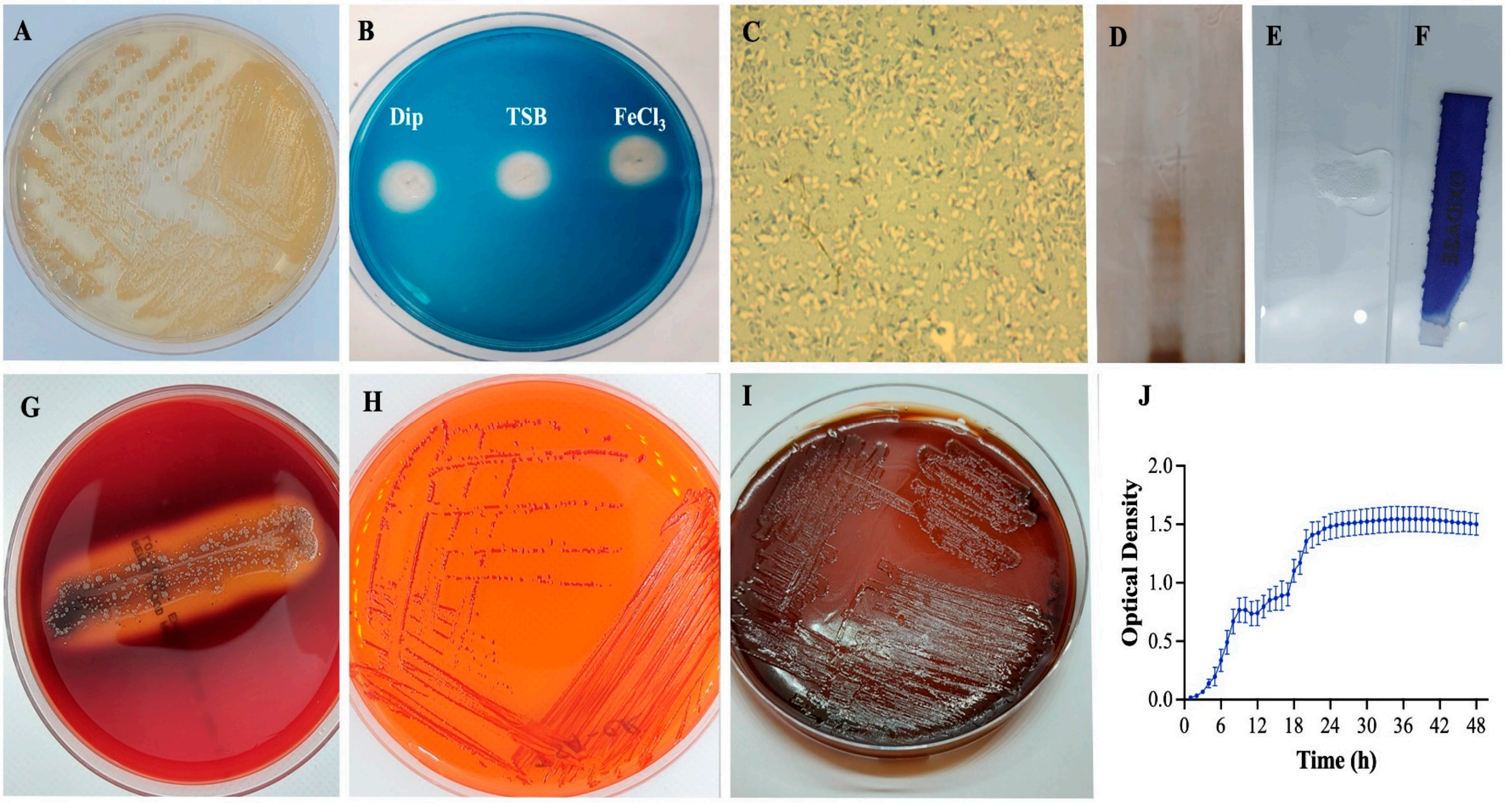

Figure S1. Phenotypic characteristics of *Vibrio* sp. J383. **(A)** Growth on TSA with 2% NaCl. **(B)** Siderophore synthesis on chrome azurol S (CAS) agar plates from *Vibrio* sp. J383 grown under iron-enriched conditions (TSB supplemented with 100 µm of $FeCl_3$), standard culture conditions (TSB), and iron limited conditions (TSB supplemented with 100 µm of 2,2-diprydil) at 15ºC. Yellow reaction around *Vibrio* sp. J383 colony indicative of being positive for siderophore synthesis. **(C)** Capsule stain of *Vibrio* sp. J383. **(D)** LPS profile for *Vibrio* sp. J383. **(E)** Positive catalase test. **(F)** Positive Oxidase test. **(G)** B hemolysin production in sheep blood agar. **(H)** Growth on congored agar. **(I)** Growth on salmon blood agar and B hemolysin production. **(J)** Growth in TSB with 2% NaCl at 15°C. Culture was aerated with aeration (180 rpm) and grown in a 12 well plate for 48 h.

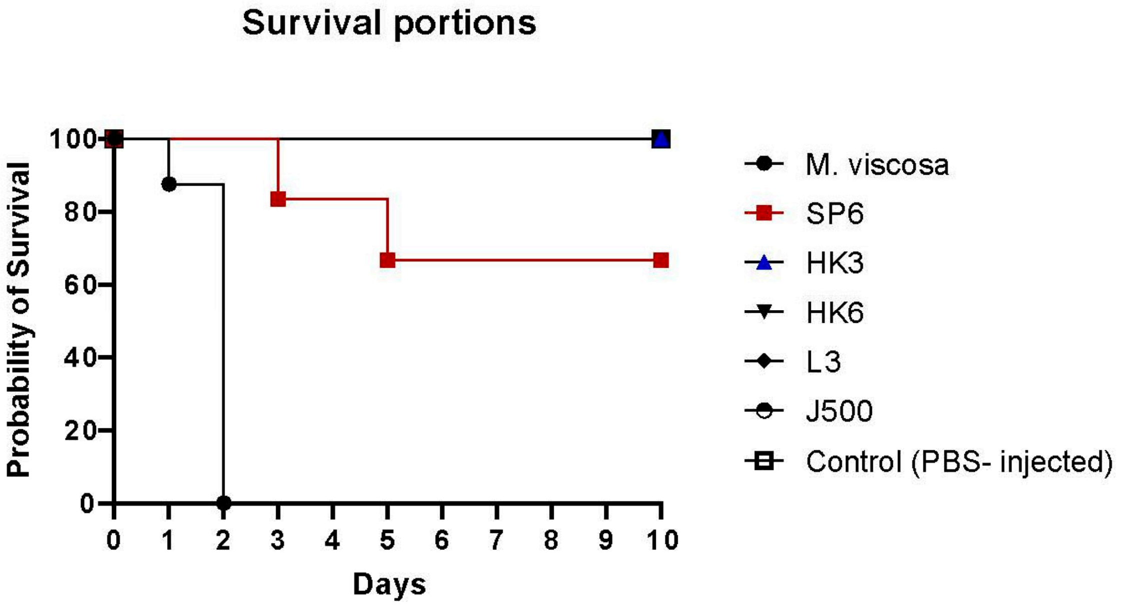

Figure S2. Acute mortality of Atlantic salmon infected with different bacterial strains isolated from fish exhibiting ‘winter ulcer’-like clinical signs. *Moritella viscosa* J311 was obtained from American Type Culture Collection, NHI. The animals were injected with 100 ul of pathogen (10^8 CFU / dose) or PBS.”

A

		1	2	3	4	5	6	7	8	9	10	11	12	13	14	15	16	17	18	19	20
Vibrio harveyi 345	1		83.58	83.53	83.61	83.28	84.80	84.41	83.86	0.00	83.48	86.26	84.12	83.95	83.98	84.40	0.00	83.47	85.79	90.08	84.57
Vibrio spp. J383 Chromosome_1	2	10.75		83.01	95.75	82.91	83.67	83.96	83.83	0.00	83.03	83.58	83.70	83.65	83.88	83.98	0.00	83.58	83.54	83.71	83.84
Vibrio cholerae O1 str	3	5.11	3.35		82.98	82.21	83.53	84.06	84.37	84.39	99.97	83.29	83.96	83.65	83.77	84.05	0.00	95.50	83.37	83.59	83.45
Vibrio splendidus BST398	4	10.90	86.53	3.54		82.87	83.86	83.98	83.85	0.00	82.99	83.61	83.58	83.47	83.84	84.02	0.00	83.07	83.50	83.76	83.84
Photobacterium damselae subsp. piscicida	5	2.95	2.47	1.83	2.53		83.11	82.71	82.64	0.00	82.21	83.25	82.80	82.78	82.75	82.71	0.00	82.39	83.42	83.16	82.96
Vibrio vulnificus FORC-077	6	13.78	7.63	8.05	7.46	2.74		84.29	84.08	0.00	83.51	84.48	84.66	84.39	84.11	84.29	0.00	83.54	85.14	84.77	95.77
Vibrio anguillarum J360	7	5.87	4.74	8.68	4.78	2.01	7.17		98.55	0.00	84.12	84.00	98.56	98.51	98.48	99.99	0.00	84.07	84.10	84.08	84.20
Vibrio anguillarum MHK3	8	5.88	4.90	9.74	4.90	2.15	7.31	84.23		0.00	84.54	83.82	98.79	98.75	98.61	98.56	0.00	84.76	83.96	83.90	83.98
Vibrio vulnificus FDAARGOS-119	9	0.19	0.11	1.13	0.13	0.02	0.09	0.60	0.44		84.39	0.00	0.00	0.00	0.00	0.00	81.70	0.00	0.00	0.00	0.00
Vibrio cholerae FDAARGOS-223	10	5.21	3.39	97.57	3.59	1.85	8.27	8.87	9.97	1.14		83.28	83.97	83.83	83.96	84.10	0.00	95.49	83.33	83.58	83.42
Vibrio alginolyticus 2439-01	11	41.47	9.97	5.53	10.03	3.01	13.94	5.71	6.31	0.28	5.63		84.03	83.98	83.96	83.98	0.00	83.45	86.68	86.43	84.49
Vibrio anguillarum CENVA NB11008	12	5.68	4.69	8.93	4.88	2.00	7.61	82.67	88.59	0.48	9.03	5.75		98.76	98.58	98.55	0.00	84.47	84.72	84.04	84.44
Vibrio anguillarum 775	13	5.69	4.61	8.36	4.86	1.98	7.44	82.07	87.82	0.37	8.57	5.93	86.46		98.52	98.54	0.00	83.83	84.79	84.00	83.79
Vibrio anguillarum JLL 237	14	5.47	4.62	8.34	4.72	2.00	6.95	82.41	87.50	0.66	8.70	5.86	84.93	87.27		98.49	0.00	84.11	84.16	83.90	84.16
Vibrio anguillarum VIB43	15	5.95	4.83	8.67	4.84	2.00	7.26	94.54	85.19	0.61	8.89	5.78	83.46	82.87	83.49		0.00	84.36	84.20	84.10	84.20
Vibrio fluvialis ATCC 33809	16	0.19	0.16	0.24	0.10	2.74E-4	0.46	0.12	0.76	1.69	0.24	0.09	0.27	0.24	0.13	0.13		0.00	0.00	0.00	0.00
Vibrio cholerae RFB05	17	4.98	3.32	83.05	3.37	1.65	8.12	8.50	9.97	0.90	84.51	5.16	9.19	8.70	9.00	8.95	0.57		84.26	83.51	83.48
Vibrio parahaemaloticus R14	18	43.81	10.16	5.82	10.45	3.04	16.04	5.74	6.20	0.31	5.93	55.78	6.69	6.90	6.16	5.88	0.22	6.32		85.75	84.62
Vibrio campbellii ATCC 25920, CAIM 519T	19	70.90	11.92	5.32	12.09	3.16	14.63	5.73	6.07	0.30	5.42	43.52	5.68	5.80	5.88	5.77	0.24	5.38	45.74		84.71
Vibrio vulnificus Env1	20	13.73	7.27	7.48	7.48	2.78	87.16	7.04	7.17	0.09	7.64	13.55	7.21	7.03	6.73	7.12	0.22	7.42	15.11	14.56	

B

		1	2	3	4	5	6	7	8	9	10	11	12	13	14	15	16	17	18	19	20
Vibrio vulnificus FDAARGOS-119	1		84.31	0.00	0.00	0.00	0.00	0.00	0.00	0.00	0.00	0.00	0.00	0.00	0.00	0.00	0.00	0.00	0.00	0.00	0.00
Vibrio fluvialis ATCC 33809	2	9.36		0.00	0.00	0.00	0.00	0.00	0.00	0.00	0.00	0.00	0.00	0.00	0.00	0.00	0.00	0.00	0.00	0.00	0.00
Vibrio harveyi 345	3	0.10	0.10		82.74	0.00	83.11	0.00	86.38	0.00	0.00	0.00	87.11	0.00	0.00	0.00	0.00	0.00	85.15	88.38	85.88
Vibrio spp. J383 Chromosome_2	4	0.08	0.05	2.69		0.00	93.31	0.00	84.15	0.00	0.00	0.00	83.36	0.00	0.00	0.00	0.00	0.00	83.58	84.07	84.11
Vibrio cholerae O1 str	5	0.63	0.20	0.10	0.15		0.00	0.00	0.00	85.15	85.08	100.00	0.00	85.60	85.23	85.46	85.15	94.10	0.00	0.00	0.00
Vibrio splendidus BST398	6	0.17	0.13	2.58	76.36	0.19		0.00	84.67	0.00	0.00	0.00	83.71	0.00	0.00	0.00	0.00	0.00	84.19	83.82	84.52
Photobacterium damselae subsp. piscicida	7	6.29E-5	3.16E-4	0.07	2.15E-4	6.37E-3	2.90E-4		0.00	0.00	0.00	0.00	0.00	0.00	0.00	0.00	0.00	0.00	0.00	0.00	0.00
Vibrio vulnificus FORC-077	8	0.05	0.32	5.44	1.14	0.85	1.14	1.50E-4		0.00	0.00	0.00	85.35	0.00	0.00	0.00	0.00	82.55	86.09	84.92	94.80
Vibrio anguillarum J360	9	0.24	0.11	0.03	0.02	2.23	0.08	8.11E-4	0.31		98.54	85.16	0.00	98.41	98.40	98.35	100.00	82.15	0.00	0.00	0.00
Vibrio anguillarum MHK3	10	0.29	0.06	0.01	0.10	2.82	0.09	9.25E-5	0.38	71.56		85.21	0.00	98.64	98.49	98.40	98.54	83.76	0.00	0.00	0.00
Vibrio cholera FDAARGOS-223	11	0.62	0.19	0.10	0.15	98.21	0.19	6.30E-3	0.84	2.21	2.82		0.00	86.00	85.24	85.47	85.17	94.10	0.00	0.00	0.00
Vibrio alginolyticus 2439-01	12	0.04	0.15	20.60	2.40	0.18	2.08	0.08	2.47	0.16	0.17	0.18		0.00	0.00	0.00	0.00	85.43	88.47	86.62	85.11
Vibrio anguillarum CNEVA NB11008	13	0.32	0.07	0.03	0.02	2.70	8.99E-3	9.19E-5	0.39	74.32	77.04	2.87	0.09		98.47	98.41	98.40	83.84	0.00	0.00	0.00
Vibrio anguillarum 775	14	0.20	0.07	0.02	0.03	2.74	0.10	0.00	0.37	75.48	80.48	2.71	0.18	80.26		98.38	98.39	83.42	0.00	0.00	0.00
Vibrio anguillarum JLL 237	15	0.44	0.06	0.01	0.09	2.73	0.09	9.04E-5	0.24	74.29	73.17	2.70	0.19	75.93	77.28		98.35	84.60	0.00	0.00	0.00
Vibrio anguillarum VIB43	16	0.28	0.11	0.03	0.02	2.25	0.08	8.17E-4	0.31	90.96	72.19	2.22	0.16	74.85	76.00	74.82		82.15	0.00	0.00	0.00
Vibrio cholerae RFB05	17	0.46	0.24	0.63	0.07	70.70	0.08	5.89E-3	1.62	1.62	2.14	69.90	1.01	2.19	2.22	2.24	1.63		83.49	0.00	81.87
Vibrio parahaemolyticus R14	18	0.09	0.25	19.34	1.95	0.17	1.83	0.08	4.20	0.18	0.32	0.16	42.46	0.09	0.18	0.09	0.18	1.32		84.24	85.98
Vibrio campbellii ATCC 25920, CAIM 519T	19	0.17	0.16	50.29	3.63	0.60	2.24	0.08	3.38	0.09	0.18	0.59	20.77	0.18	0.10	0.09	0.09	0.52	17.20		85.22
Vibrio vulnificus Env1	20	0.05	0.23	4.26	1.15	0.89	1.18	7.66E-5	86.10	0.27	0.35	0.87	2.44	0.35	0.30	0.35	0.27	1.04	4.14	2.86	

Figure S3. Average nucleotide identity in *Vibrio* sp. J383. **(A)** chromosome 1; **(B)** chromosome 2. *Vibrio*. sp. J383 identity percentage parameters for annotated genes were set up as minimum similarity of 0.8 and minimum length of 0.8. Analyses were performed using CLC Genomic Workbench v20 (CLC Bio). Genome alignment involved 20 *Vibrio* sp.
