# Supplementary material for: Comparative Genomic Analysis of a Novel Vibrio sp. Isolated from an Ulcer Disease Event in Atlantic Salmon (Salmo salar)
Source: Microorganisms. 2023 Jul 2;11(7):1736. doi: 10.3390/microorganisms11071736 (PMC10385127; doi:10.3390/microorganisms11071736)
Supplement: Supplementary file 1 [file microorganisms-11-01736-s001.zip › Article 1- Supplementary Tables .pdf]

Table S1. Enzymatic profile of *Vibrio*. sp. J383 using commercial biochemical test kits (API system).

APIZYM		API20E		API20NE	
Alkaline phosphatase	+	β-Galactosidase	+	Reduction of nitrates to nitrites	+
Esterase (C4)	+	L-Arginine	-	Indole production	+
Esterase lipase (C8)	+	L-Lysine	-	Glucose fermentation	+
Lipase	+	L-Ornithine	-	Arginine	-
Leucine arylamidase	+	Citrate utilization	-	Urease	-
Valine arylamidase	+	H2S production	-	Esculin	+
Cystine arylamidase	+	Urease	-	Gelatin	+
Trypsin	+	L-Tryptophane	+	β-galactosidase	+
Chymotrypsin	+	Indole production	+	D-Glucose	+
Acid phosphatase	+	Acetoin production	-	L-Arabinose	-
Naphthol-AS-BI-phosphohydrolase	+	Gelatinase	+	D-Mannose	-
Galactosidase	+	D-Glucose	+	D-mannitol	+
B- galactosidase	-	D-Mannitol	+	N-acetyl-glucosamine	-
Glucuronidase	-	Inositol	-	D-maltose	-
B-glucuronidase	-	D-Sorbitol	-	Potassium Gluconate	-
Glucosidase	-	L-Rhamnose	-	Capric acid	-
B-glucosidase	-	D-Saccharose	-	Adipic acid	-
N-acetyl-B-glycosaminidase	-	D-Melibiose	-	Malic acid	-
Mannosidase	-	D-Amygdaline	+	Trisodium citrate	-
Fucosidase	-	L-Arabinose	-	Phenylacetic acid	-

Table S2. Genes associated with subsystems in chromosomes and plasmid

Predicted genes associated with subsystems pathogenesis and environmental adaptations			
**Gene Subsystem Category or Gene Name**	**Presence/Absence of Gene in *Vibrio* sp. J383**		
	**Chromosome 1**	**Chromosome 2**	**Plasmid**
MazEF toxin-antitoxing			+
Flagellum	+		
Fatty acid biosynthesis	+		
Ferrichrome-iron receptor	+		
fur	+		
mdtL		+	
fabG	+		
Vibrio Ferrin synthesis	+		
Lipoic acid metabolism	+	+	
Polyamine metabolism	+	+	
Cold shock protein of CSP family		+	
Lactose utilization		+	
DNA repair, bacterial photolyase	+	+	
Cell division cluster	+		
Antibiotic targets in DNA processing	+		
Resistance to chromium compounds	+		
Xanthine dehydrogenase subunit		+	
Thiamin, thiazole, hydroxyethyl pyrimidine uptake		+	
Bacterial checkpoint control related cluster	+		
Proteasome bacterial	+		
Chemotaxis	+		
Antibiotic targets in cell wall biosynthesis	+	+	
Heme transport		+	
Iron ABC transport permease		+	
Toxin antitoxin system		+	
Antibiotic target in transcription	+		
